# When One Shape Does Not Fit All: A Commentary Essay on the Use of Graphs in Psychological Research

**DOI:** 10.3389/fpsyg.2017.01666

**Published:** 2017-09-25

**Authors:** Massimiliano Pastore, Francesca Lionetti, Gianmarco Altoè

**Affiliations:** ^1^Department of Developmental and Social Psychology, University of Padova Padova, Italy; ^2^Department of Biological and Experimental Psychology, Queen Mary University of London London, United Kingdom

**Keywords:** statistical reasoning, bar chart and box plot, graphical representation, exploratory data analysis, credibility crisis

## 1. Introduction

The interest of psychology in graphs is anything but new. It was 1972 when John Wilder Tukey, one of the fathers of the statistic of the XX century, distinguished among three categories of graphs: (1) *propaganda graphs*, that are intended to show what already can be learned using data-analysis and inferential testing, (2) *analytical graphs*, that allow to understand data over and above what inferential statistic has already shown, and (3) the *substitute for tables*, that are graphs from which numbers are to be read off (Tukey, 1977). From this classification it appears evident Tukey's recommendation for analytical graphs. Twenty years later, Leland Wilkinson and the Task Force on Statistical Inference of the American Psychological Association, similarly posited: “Before you compute any statistics, look at your data. (…) If you assess hypotheses without examining your data, you risk publishing nonsense” (Wilkinson and Task Force on Statistical Inference, 1999). Nowadays, visual inspection continuous to be largely recommended for understanding data set's meaning in exploratory data analysis, and is considered more useful than a solely strictly adherence to statistical testing to answer questions prompted by the experiment (Wixted and Pashler, 2002; Marmolejo-Ramos and Matsunaga, 2009). Also student's books and papers addressing mechanisms underpinning statistical reasoning have introduced a shift of perspective from *drawing* graphs to *using* graphs for making sense of data and evaluating hypotheses (Moore, 1998; Wild and Pfannkuch, 1999; Konold and Pollatsek, 2002; Bakker, 2004; Bakker and Gravemeijer, 2004; Pfannkuch, 2005; Watson, 2005; Garfield and Ben-Zvi, 2008; Matejka and Fitzmaurice, 2017). However, as we review below, a vast majority of research papers continue to adopt non optimal graphical representations. Also, though graphs could make data transparent, increasing the reliability of research findings (Tay et al., 2016), among guidelines proposed for promoting transparency in research (Nosek et al., 2015), no specific reference is made upon the relevance of adequate graphical representations.

## 2. Static graphs and bar charts

A recent systematic review (Weissgerber et al., 2015) of research articles published in top physiology journals in 2014 showed that the most often used graphical representations are *static graphs* and, among these, the widely known bar chart. Bar charts, useful for depicting frequencies and the occurrence of categorical variables, summarize means and standard deviations without depicting the underlying distribution of data. It results that nothing else is provided beyond what already the statistics show, increasing the risk to misinterpret research findings and to not detect important information (Cooper et al., 2002; Schriger et al., 2006; Saxon, 2015; Gelman, 2017). For example, the presence of anomalous outliers or of marked asymmetry cannot be inferred. Though a systematic review on this topic has not been published yet in psychology, research articles published between January and June 2016 in four high impact psychology journals (*Behaviour Research Methods, Cognitive Psychology, Psychological Science*, and *Trends in Cognitive Science*) suggest a state of the art that does not differ much from that of other disciplines using statistical methods, with a significant presence of bar chart graphs in a field of research where continuous variables (e.g., reaction times, psychological test scores) are almost the norm (see also Bar Bar Plots Project, 2017). Specifically, on 131 research papers examined, bar charts were about 55% of 104 presented graphs.

To explain the impact of inadequate graphs more clearly and to understand the practical implications of this, we provide three examples below. To make our examples clear, we designed the three vignettes so that there is always a comparison between two experimental conditions and the analysis of variance (ANOVA) is used to statistically compare groups. The exemplifications that we provide show that an adequate use of graphs as a reasoning tool leads to results that differ from those that would have been reached if only summary statistics and static bar chart would had been adopted. We conclude that appropriate graphical representations can increase reliability in research findings and promote transparency in the way scientific information is shared and disseminated.

## 3. The three vignettes

### 3.1. Example 1: the hidden difference

Let's suppose that 200 students are recruited from two different classes (*a, b*) and randomly assigned by a researcher to two independent experimental conditions (*x, y*). In each condition, subjects' performance on a specific experimental task is assessed. The research hypothesis is that there is a significant difference between the two experimental conditions.

**Figure 1:**
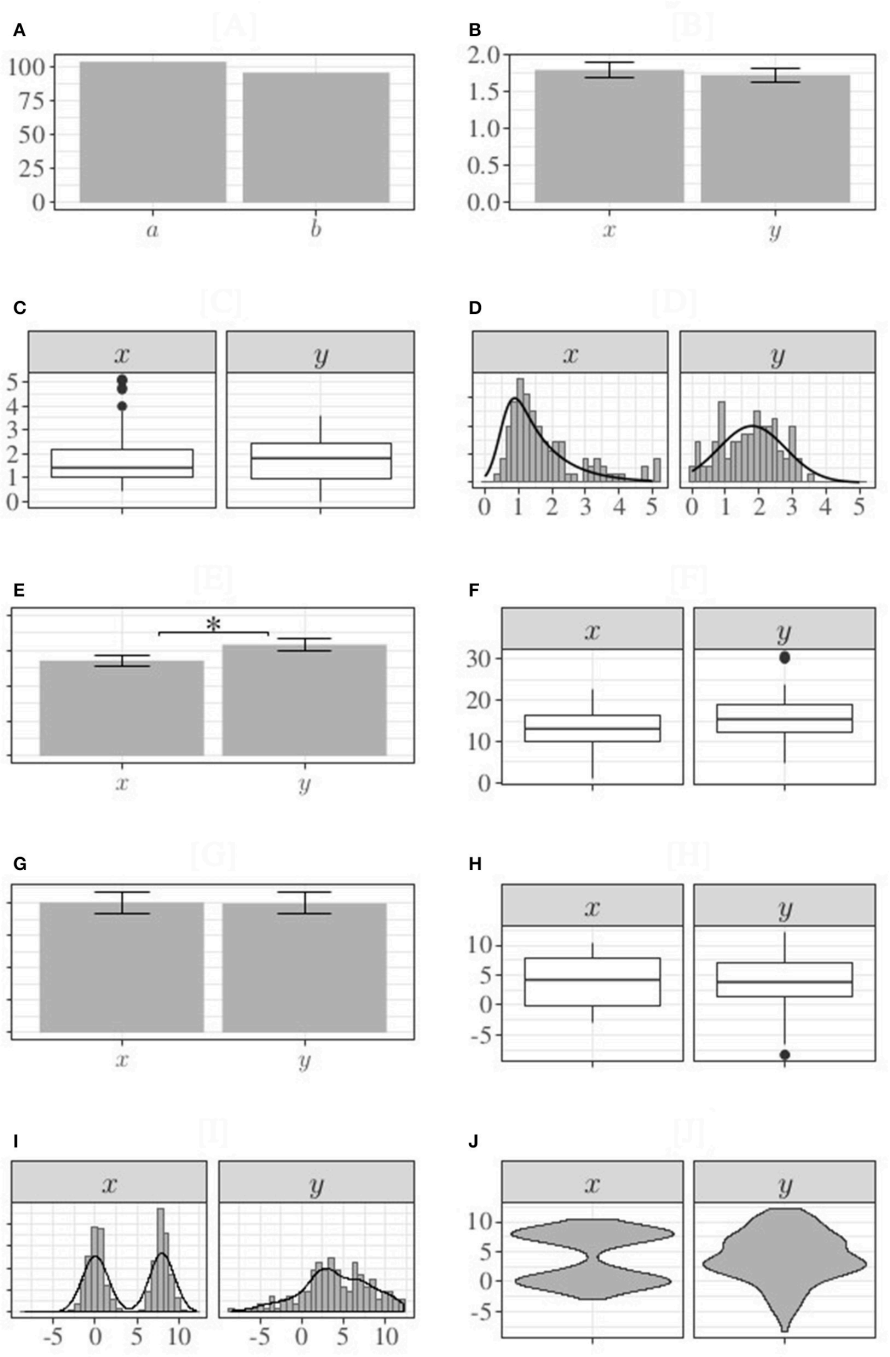
Graphical representation of examples provided in the text. **(A–D)** Refer to example 1, section The Hidden Difference; **(E,F)** refer to example 2, section The Feigned Difference; **(G–J)** refer to example 3, section When One Graph Does Not Fit All. ^*^Indicates a significant difference between means at 0.05 level.

In Figure 1A, number of subjects belonging to each class is depicted: 104 subjects belong to class *a*, and 96 to class *b*. The height of each bar correctly represents frequency of subjects in each condition and, therefore, bar chart is informative and pertinent. In Figure 1B, using the same graph, subjects' mean scores (and associated standard errors) in condition *x* and *y* are depicted: 1.8 and 1.73 respectively. Contrary to the research hypothesis, bar chart suggests no difference between the two experimental conditions, as confirmed also by ANOVA: *F*_(1, 198)_ = 0.24, *p* = 0.63, Cohen's *d* = 0.07.

Representing the same data using a box plot (Figure 1C) and a histogram (Figure 1D), we end up with a different conclusion. Condition *x* shows a skewed distribution while, on the contrary, data from condition *y* are more symmetrically distributed, suggesting that the two experimental conditions are not equivalent. The research hypothesis is now supported. Without the use of graphs as reasoning tools for exploring data, it would had never been possible to detect this difference in the two experimental conditions and the researcher would had not supported, inappropriately, his research hypothesis.

### 3.2. Example 2: the Feigned difference

Let's consider now a second example, in which we compare again two experimental conditions, but this time with less subjects per each (small samples are common in experimental psychology) and, specifically, 50. Mean scores and standard errors are depicted in the bar chart in Figure 1E. Bar chart suggests that the two experimental conditions are different. Similarly, also ANOVA results suggest a significant difference between the two conditions: *F*_(1, 98)_ = 4.38, *p* = 0.04, Cohen's *d* = 0.42. When the same data are depicted using a box plot in the place of a bar chart, the presence of outliers appears evident (Figure 1F). Excluding these outlier values, the difference between the two experimental conditions is not significant anymore: *F*_(1, 95)_ = 1.87, *p* = 0.17, Cohen's *d* = 0.28. In relative small sample size studies, outliers may strongly influence statistical results and can be easily identified using adequate graph representations.

### 3.3. Example 3: when one graph does not fit all

In the two examples reported above, the use of bar charts did not allow to properly detect false negative (example 1) and false positive (example 2) research findings. In both cases, adopting two alternative graph options (box plot for summarizing data, and histogram for plotting individual values) it was possible to accurately explore pattern of data that were otherwise concealed by bar charts. Is box plot the best graphical option able to adequately fit any type of data? Unfortunately, this is not the case. Box plots, more informative than bar charts for representing summary statistics of continuous variables, may fail as well in specific conditions, as we discuss in the current example.

Let's consider again two experimental conditions: means and standard errors are depicted in Figure 1G. It's easy to see that mean values (and standard errors) are comparable [see also *F*_(1, 598)_ = 0.01, *p* = 0.94, Cohen's *d* = 0.01]. When a box plot is adopted in the place of a bar chart, a difference in variability emerged (see Figure 1H). Using a histogram for further exploring the pattern of data (see Figure 1I) a bimodal distribution in the condition *x* becomes easily identifiable. Box plots, more informative than bar charts (as we demonstrated with Example 1 and Example 2), may be not enough for gaining full information of data because do not allow to detect multimodal distribution. In this case the more adequate representation is the violin plot (see Figure 1J). Such type of graph, including information about densities of the distributions, allow to detect even multimodal distribution of data.

## 4. Concluding remarks

Graphical representations are useful to become familiar with and understand the concept of variation, as well as to investigate the sources and the impact of variance on observed data, which are among the main aims of psychological research. Graphs remind us that the process of statistical inference is not mechanical (Gigerenzer and Marewski, 2015; McElreath, 2016). This process often involves subjective decisions (e.g., the evaluation, exploration, and/or deletion of outliers) which are an integral part of the analysis. Thus, graphs are among the most appropriate tools to enhance transparency and confer plausibility to such decisions.

As we demonstrated with three simple examples, an accurate visual representation of data plays a pivotal role in the interpretation of research findings, representing a truly inferential statistic tool. Bar chart graph does not allow to fully explore data distribution, and may conceal important information increasing the risk to publish unreliable findings that fail at replication. Also, not adding anything more than what already summary statistics show, bar chart is of limited utility for promoting statistical reasoning on data, increasing the risk of a mechanical approach to data analysis. Widely adopted in psychology, bar chart is useful for depicting frequencies and categorical variables but may be misleading if adopted to represent summary statistics of continuous variables, as a companion of *t*-test and ANOVA. Histograms, allowing to depict the distribution of all data, are recommended, but do not offer the opportunity of summarizing data in a clear and effective way. Other graphical representations, more informative but less disseminated in our field, as box plot and violin plot, could represent a more informative option. More generally, combining various graphical techniques could allow researchers to know more about full data, and promote the access to relevant information otherwise concealed by static data graphs. Improved data representation techniques could be one of the way for enhancing students' understanding of statistical reasoning, the scientific community understanding of published data, and a critical evaluation of research findings. Also, making data transparent, graphs may represent one of the answers to the crisis of credibility in psychology (see: Ioannidis, 2005; Pashler and Wagenmakers, 2012; Open Science Collaboration, 2015). The increased availability of powerful statistical software has opened the possibility of using new and more sophisticated analytic approaches, which are testified by recent scientific publications. However, this progress has yet to be fully integrated in graphical representations of data.

## Author contributions

MP proposed the topic and designed the vignettes; FL and GA contributed to further develop the idea; MP, FL, and GA wrote the paper.

### Conflict of interest statement

The authors declare that the research was conducted in the absence of any commercial or financial relationships that could be construed as a potential conflict of interest.
